# Association Study of *TAF1* Variants in Parkinson’s Disease

**DOI:** 10.3389/fnins.2022.846095

**Published:** 2022-04-08

**Authors:** Qian Zeng, Hongxu Pan, Yuwen Zhao, Yige Wang, Qian Xu, Jieqiong Tan, Xinxiang Yan, Jinchen Li, Beisha Tang, Jifeng Guo

**Affiliations:** ^1^Department of Neurology, Xiangya Hospital, Central South University, Changsha, China; ^2^Key Laboratory of Hunan Province in Neurodegenerative Disorders, Central South University, Changsha, China; ^3^National Clinical Research Center for Geriatric Disorders, Xiangya Hospital, Central South University, Changsha, China; ^4^Hunan Key Laboratory of Medical Genetics, School of Life Sciences, Centre for Medical Genetics, Central South University, Changsha, China; ^5^Department of Geriatrics, Xiangya Hospital, Central South University, Changsha, China; ^6^Hunan International Scientific and Technological Cooperation Base of Neurodegenerative and Neurogenetic Diseases, Changsha, China

**Keywords:** Parkinson’s disease, *TAF1* variants, coding variants, chinese population, whole-exome sequencing

## Abstract

Increasing evidence reveals sex as an important factor in the development of Parkinson’s disease (PD), but associations between genes on the sex chromosomes and PD remain unknown. *TAF1* is a gene located on the X chromosome which is known to cause X-linked syndromic mental retardation-33 (MRXS33) and X-linked Dystonia-Parkinsonism (XDP). In this study, we conducted whole-exome sequencing (WES) among 1,917 patients with early-onset or familial PD and 1,652 controls in a Chinese population. We detected a hemizygous frameshift variant c.29_53dupGGA(CAG)_2_CTACCATCA(CTG)_2_C (p.A19Dfs*50) in two unrelated male patients. Further segregation analysis showed an unaffected family member carried this variant, which suggested the penetrance of the variant may be age-related and incomplete. To verify the effects of *TAF1* on PD, genetic analyses were carried separately by gender. Analysis of rare variants by optimal sequence kernel association (SKAT-O) test showed a nominally significant difference in variant burden between the male PD patients and controls (2.01 vs. 1.38%, *p* = 0.027). In the female group, none of the variant types showed significant association with PD in this study. In conclusion, we found rare variants in *TAF1* may be implicated in PD, but further genetic and functional analyses were needed.

## Introduction

Parkinson’s disease (PD) is a complex neurodegenerative disorder characterized mainly by nigrostriatal cell loss and Lewy body formation (Belarbi et al., 2020). Sex is an important factor in the development of PD, as reflected by the fact that PD is more common in men than in women (Jurado-Coronel et al., 2018). *TAF1* encodes TATA-binding protein-associated factor-1, which plays a key role in transcriptional initiation (Bragg et al., 2019). The *TAF1* gene locates on the X chromosome and includes 38 exons, along with five additional exons located 3′ to exon 38 (exons d1–d5) (Domingo et al., 2016). Combinations of these exons constitute a multiple transcript system that is alternatively spliced (Muller et al., 2007). In 2015, O’Rawe et al. (2015) found mutations in *TAF1* were associated with X-linked syndromic mental retardation-33 (MRXS33), and knockdown of this gene in zebrafish resulted in a 10% reduction in the relative area of the optic tectum, suggesting a neuronal defect. Besides, recent studies have finally confirmed that X-linked Dystonia-Parkinsonism (XDP) was caused by SINE-VNTR-Alu (SVA) retrotransposon insertion in intron 32 of the *TAF1* gene (Aneichyk et al., 2018). XDP is a rare neurological disease found predominantly in men from the island of Panay, Philippines (Lee et al., 2011). Most of the XDP patients at the advanced stages of disease progression shift to symptoms comparable to those found in PD, such as bradykinesia and tremor (Lee et al., 2002). Interestingly, 14% of cases present initially with parkinsonism (Pauly et al., 2020). In some XDP patients, single-photon emission computed tomography (SPECT) images showed functional disturbance of nigrostriatal projections (Bruggemann et al., 2017), and loss of dopaminergic neurons in nigrostriatal pathway was also involved in PD patients (Qian et al., 2020).

However, there was still a lack of comprehensive research to investigate the involvement of *TAF1* variants in PD. Here, we reported clinical and genetic findings of two unrelated families with a frameshift variant in *TAF1*. To elucidate the role of *TAF1* in PD, we performed a systematic survey in a Chinese mainland population with PD using whole-exome sequencing (WES).

## Materials and Methods

### Subjects

As described in our previous study (Zhao et al., 2021), we enrolled PD patients from Xiangya Hospital and other cooperating centers of Parkinson’s Disease and Movement Disorders Multicenter Database and Collaborative Network in China (PD-MDCNC).^1^ PD was diagnosed according to the United Kingdom Parkinson’s Disease Society Brain Bank criteria (Hughes et al., 1992) or Movement Disorders Society (MDS) clinical diagnostic criteria (Postuma et al., 2015). Healthy controls of Chinese ancestry enrolled from communities did not have any neurological or psychiatric system diseases. Only PD patients with age at onset (AAO) no more than 50 years or with a family history of PD were included. This study was approved by the Ethics Committee of Xiangya Hospital (Central South University), and all study subjects provided written informed consent. Genomic DNA was extracted from peripheral blood leukocytes.

### Genotyping and Quality Control

Briefly, WES was performed using Agilent SureSelect Human All Exon Kit V6. Paired-end reads were generated by Illumina X10 platform with a read length of 2 × 150 bp, average depth of 123-fold. The reads were mapped to the human reference genome (hg19) with Burrows-Wheeler Aligner (BWA) (Li and Durbin, 2009), and duplicate reads were removed with Picard Tools.^2^ Next, Genome Analysis Toolkit (GATK) was used for base quality-score recalibration (BQSR) and local realignment around insertions/deletions (indels) (McKenna et al., 2010). The remaining variants were further annotated with ANNOVAR (Yang and Wang, 2015) and VarCards (Li et al., 2018a) based on RefSeq (hg19) for gene regions, amino acid alterations, functional effects and allele frequencies in the East Asian population [Genome Aggregation Database (gnomAD); Exome Aggregation Consortium (ExAC) database]. ReVe (Li et al., 2018b) was run to predict the functional effects of the variants (threshold, 0.7).

Quality-control procedures were carried out with PLINK [version 1.90 (Christopher Chang/Grail Inc.)] (Chang et al., 2015). We excluded samples with discordant sex information or pathogenic/likely pathogenic variants of PD disease-causing genes, deviating heterozygosity/genotype calls (±3 standard deviations), low genotype call rates (missing rate > 10%) or cryptic relatedness (identity by descent > 0.15). Variants with low-quality genotypes [Phred-scaled genotype quality score (GQ) below 20, allele depth (AD) below 5, and reads depth (DP) below 10], missing genotype rate greater than 0.1, or deviating significantly (*p* < 0.0001) from Hardy-Weinberg equilibrium were also filtered. We performed principal-component analysis (PCA) and sample outliers (non-Chinese ancestry) were filtered. Of note, the X-chromosome variants underwent additional quality control steps. X-specific quality control included filtering variants that were not in Hardy-Weinberg equilibrium in female controls (*p* < 0.0001), that had significantly different minor allele frequency (MAF) or missingness rates between males and females in control individuals (*p* < 0.05/number of variants), as well as removal of the pseudoautosomal regions.

### Sanger Sequencing Validation

A frameshift variant of *TAF1* detected in two unrelated families was validated by Sanger sequencing. Oligonucleotide primers were designed with Primer3 program.^3^ Polymerase chain reaction (PCR) analysis of the *TAF1* gene was carried out by using 2 pairs of primers. Detailed methods and primer sequences were provided in the Supplementary Material and Supplementary Table 1.

### Statistical Analysis

Fisher’s exact test in R software (version 3.6.3) was conducted to test the significance of differences in frequencies between different groups. In addition, variants within the coding regions (chrX:70,586,225-70,683,896, NM_004606, GRCh37/hg19) (Zweig et al., 2008) were extracted for further analysis. Then we used a 1% MAF threshold to define rare and common variants for all samples. ReVe was used to predict the pathogenicity of variants (threshold, 0.7). *TAF1* gene was located on the X chromosome so variants were analyzed separately by sex. Gene-based analysis was conducted by the Optimal Sequence Kernel Association (SKAT-O) (Lee et al., 2012), adjusting for age/AAO (age for controls; AAO for cases) and the first five principal components. Because we have performed multiple interdependent analyses, we used a false discovery rate (FDR) correction for multiple comparisons with an FDR-corrected *p* values < 0.05 considered as statistically significant.

## Results

### Study Population

Our study included 1,917 participants with PD [1,047 men (54.6%); mean (SD) age, 52.2 (9.0) years] and 1,652 healthy controls [795 men (48.1%); mean (SD) age, 62.0 (12.6) years]. PD patients had a mean AAO of 46.3 years (SD = 8.4). The demographic data for our study were given in Table 1.

**TABLE 1:** Demographic data of the study population.

Clinical features	Case (*n* = 1,917)	Control (*n* = 1,652)
Sex, male (*n*, %)	1,047, 54.6%	795, 48.1%
Age (years, mean ± SD)	52.2 ± 9.0	62.0 ± 12.6
Age at onset (years, mean ± SD)	46.3 ± 8.4	

*n, sample size; SD, standard deviation.*

### Identification of the Frameshift Variant

The WES analysis revealed a hemizygous duplication of 25 bp within 5′ UTR of the *TAF1* gene in two unrelated male patients [c.29_53dupGGA(CAG)_2_CTACCATCA(CTG)_2_C], which is predicted to cause frameshift for 49 amino acids, and premature stop at codon 68 (p.A19Dfs*50) (Supplementary Figure 1B). This variant was considered as uncertain significance by the American College of Medical Genetics and Genomics (ACMG) criteria. Clinical characteristics were described in the Supplementary Materials. To study the segregation of the variant detected, sequencing of additional family members was conducted. In family 1, heterozygosity of the same variant was identified in maternal DNA (Supplementary Figure 1A), indicating that the variant was inherited from his carrier mother, and p.A19Dfs*50 variant was also present in a healthy male relative (II:3). Besides, this variant did not find in the proband’s healthy brother in family 2. The identification of p.A19Dfs*50 in an unaffected family member suggested that the penetrance of this variant is perhaps age-related and incomplete. Using Fisher’s exact test, we found no significant differences in the frequencies of the variant between the 1,917 cases and 1,652 controls (*p* = 0.502; Table 2). Similar results were found when compared the frequencies between cases and databases. Therefore, the pathogenic role of this variant still needs more validation.

**TABLE 2:** The frameshift variant in different databases.

Hg19 position	dbSNP ID	Protein Alteration	MAF in 1,917 cases	MAF in 1,652 controls	MAF in gnomAD (East Asian)	MAF in ExAC (East Asian)	*p* Value (cases-controls)	*p* Value (cases- gnomAD	*p* Value (cases- ExAC
X:70586186	rs753316021	p.A19Dfs*50	0.000728	0	0.000812	0.000756	0.502	1	1

*MAF, minor allele frequency; gnomAD, Genome Aggregation Database; ExAC, Exome Aggregation Consortium.*

### Burden Analysis of Rare Variants

**FIGURE 1:**
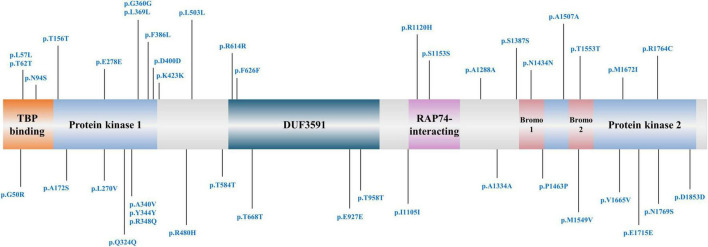
Rare coding variant sites of *TAF1* gene included in our study.

In our study, none of the common variants within coding regions were identified. Coding regions contained 42 rare variants after quality control (Figure 1 and Supplementary Table 2). All rare variants within coding regions were synonymous or missense. By *in silico* prediction with ReVe, 3 damaging missense variants were included (p.A340V, p.R480H, and p.M1549V). Rare variants in *TAF1* gene showed a suggestive association in the male group (2.01 vs. 1.38%, *p* = 0.027), but no statistically significant differences were identified after FDR correction (Table 3). In the female group, none of the variant types showed significant association with PD in this study.

**TABLE 3:** Gene-based association of *TAF1* coding variants.

Gender	Variant type	No. of variants	Case	Control	*p*_(SKAT–o)_	*p* Value*
Male	All	20	21 (2.01%)	11 (1.38%)	**0.027**	0.151
	Synonymous	14	14 (1.34%)	7 (0.88%)	**0.043**	0.151
	Missense	6	7 (0.67%)	4 (0.50%)	0.077	0.180
	Dmis	–	–	–	–	–
Female	All	32	37 (4.25%)	28 (3.27%)	0.288	0.403
	Synonymous	22	28 (3.22%)	18 (2.10%)	0.143	0.250
	Missense	10	9 (1.03%)	10 (1.17%)	0.759	0.759
	Dmis	3	1 (0.11%)	2 (0.23%)	0.549	0.641

*SKAT-O, optimal sequence kernel association test; Dmis, damaging missense (ReVe > 0.7).*

*p values shown were after false discovery rate correction.

## Discussion

In the present study, a hemizygous frameshift variant c.29_53dupGGA(CAG)_2_CTACCATCA(CTG)_2_C (p.A19Dfs*50) in two unrelated male patients were observed. Then we conducted a systematic large cohort analysis of *TAF1* variants using WES. Genetic analyses were conducted separately by sex in consideration of its cytogenetic location. For the coding regions, rare variants showed a nominally significant enrichment in male patients with early-onset or familial PD. *TAF1* encodes the largest subunit of the transcription factor II D (TFIID), which mediates transcription by RNA polymerase II (Thomas and Chiang, 2006). Previous studies have established the genetic basis of XDP, which is thought to be the SVA insertion within the *TAF1* gene (Aneichyk et al., 2018). Besides, five disease-specific single-nucleotide changes (DSCs) within *TAF1/DYT3* multiple transcript system were only found in XDP patients. Notably, Herzfeld et al. (2013) have found DSC3 in exon d4 plays an important role in the regulation of genes involved in dopamine processing and function, such as *SNAP25*, *DBH*, and *GCH1*. Additionally, DSC3 also affects d2-d4-mediated regulation of genes protective against reactive oxygen species (ROS). PD neuropathology is characterized by the loss of dopaminergic neurons in the substantia nigra pars compacta (SNc) (Zhang et al., 2018), and uncontrolled ROS generation is one of the potential causative factors for the death of dopaminergic neurons in PD (Trist et al., 2019). Up until now, no *TAF1* variants have been reported in PD patients. Here, we reported a hemizygous duplication within 5′ UTR of the *TAF1* gene in two unrelated male patients with early-onset PD by performing analysis of WES. The 5′ UTR sequences could regulate expression of genes and may be involved in protein translation or disease pathogenesis (Orom et al., 2008). Moreover, 5′ UTR regions have potential effects on mRNA translation efficiency resulting in altered protein levels (Robert and Pelletier, 2018). In this study, this frameshift variant was not detected in our control population. The MAFs in the gnomAD and ExAC databases were 0.000812 and 0.000756, respectively. Segregation analysis by sanger sequencing also showed an unaffected family member carried this variant, which suggested the pathogenic role of this variant remains uncertain. This manuscript explores whether variants in the *TAF1* region contribute to PD for the first time to our knowledge. However, UTRs, intronic and regulatory regions of *TAF1* were not under the scope of the analysis. Whole-genome sequencing studies will be required to study these regions.

As many statistical tools are now available to analyze the X chromosome, the role of gender in genetic architecture should not be ignored. In most studies, all X-chromosome variants have been removed after the quality control procedures. To consider differences in genotyping between hemizygous males and diploid females, we have performed quality control steps separately for males and females. Due to the haploid nature of males, the power to identify the association will be limited. *TAF1* gene is the causative gene of MRXS33 and XDP, and it’s noteworthy that these two diseases were observed predominantly in males (Westenberger et al., 2013; O’Rawe et al., 2015). Similarly, our results indicated a potential role for rare variants of *TAF1* gene in the male early-onset or familial PD etiology. The lack of association of *TAF1* variants in female PD patients may be caused by the pattern of X-chromosome inactivation (XCI). XCI on female X-chromosome loci is the process in which one of the X chromosomes in females is randomly inactivated to equalize the impact of the presence of two X chromosomes in females (Wang et al., 2014). Genotypes are coded by PLINK on the basis of the hypothesis that the effect of the deleterious allele in males equals the effect of the heterozygote genotype in females. Although PLINK accounts for escape from XCI, it ignores random and skewed XCI mechanisms. Due to the limitation of XCI and PLINK regression approach, more studies in different cohorts with more powerful tools are important.

Interactions between gender and genetics have been described in other complex traits such as juvenile idiopathic arthritis and schizophrenia (Shifman et al., 2008; Haasnoot et al., 2018). A previous study focused on *SRY* gene, which was located in the Y chromosome, found *SRY* variants were not associated with the risk of PD (Pan et al., 2021). To better comprehend the genetic architecture of PD, future genetic studies could take sex-stratification into account. In conclusion, although our results suggest that rare variants in *TAF1* may be implicated in PD in a Chinese mainland population, larger sample size studies and validation studies in other populations are needed to confirm the results.

## Data Availability Statement

The data have been deposited into CNGB Sequence Archive (CNSA) of China National GeneBank DataBase (CNGBdb) with accession number CNP0002638. Data of this project can be accessed after an approval application to the China National Genebank (CNGB, https://db.cngb.org/cnsa/).

## Ethics Statement

The studies involving human participants were reviewed and approved by Ethics Committee of Xiangya Hospital (Central South University). The patients/participants provided their written informed consent to participate in this study.

## Author Contributions

QZ carried out genetic analysis and wrote the manuscript. HP, YZ, and YW processed genomic data. QX, JT, XY, and JL helped the study design and data interpretation. BT and JG critically revised the manuscript. All authors contributed to the article and approved the submitted version.

## Conflict of Interest

The authors declare that the research was conducted in the absence of any commercial or financial relationships that could be construed as a potential conflict of interest.

## Publisher’s Note

All claims expressed in this article are solely those of the authors and do not necessarily represent those of their affiliated organizations, or those of the publisher, the editors and the reviewers. Any product that may be evaluated in this article, or claim that may be made by its manufacturer, is not guaranteed or endorsed by the publisher.

## Footnotes

1
http://pd-mdcnc.com

2
http://broadinstitute.github.io/picard/

3
https://bioinfo.ut.ee/primer3/
