# Supplementary material for: Association Study of TAF1 Variants in Parkinson’s Disease
Source: Front Neurosci. 2022 Apr 8;16:846095. doi: 10.3389/fnins.2022.846095 (PMC9024305; doi:10.3389/fnins.2022.846095)
Supplement: Supplementary file 1 [file Table_1.docx]

Supplementary Material

# Supplementary Methods

Sanger sequencing validation

Sanger sequencing was used to validate the frameshift variant found in the WES analysis of the patients and their families. Primers were designed with Primer3 program (https://bioinfo.ut.ee/primer3/). Polymerase chain reaction (PCR) analysis was carried out by using 2 pairs of primers and primer sequences were listed in **Supplementary Table 1**. The amplification reactions were carried out on an T100 Thermal Cycler (Bio-Rad, USA). PCRs were performed in 50 μl reaction volumes containing 45 μl of GoldenStar® T6 Super PCR Mix (green) (Tsingke Biological Technology, China), 2 μl of each 10 μM primer pair and 1 μl of DNA template. The PCR conditions were as follows: 95°C for 5 min; 15 cycles of 95°C for 45 s, 65°C for 45 s (−1°C per cycle), and 72°C for 45 s; 20 cycles of 95°C for 45 s, 50°C for 45 s, and 72°C for 45 s; and then 72°C final extension for 10 min. After amplification, products were purified and sequenced on DNA sequencing system (Applied Biosystems, 3730XL). Sequence alignment was performed using the SeqMan program in DNASTAR software (DNASTAR Inc., USA).

# Clinical characteristics of the two probands

Case 1 (Family 1, II:4) was a 49-year-old man. The patient had unilateral, tremor-predominant, levodopa-responsive PD from age 34 years. His medications included levodopa/benserazide 62.5 mg three times daily, and piribedil 50 mg. Later on, he felt limb stiffness became worse over time. At the age of 43, he underwent a Deep Brain Stimulation (DBS) operation. After the operation, he consulted our department with complaints of slow irregular speaking rhythm. He also had rapid eye movement (REM) sleep behavior disorder (RBD). Other non-motor symptoms and autonomic signs were not found in the patient. The score of the Unified Parkinson's disease rating scale (UPDRS-III motor scale) was 9/108 and Hoehn and Yahr (H&Y) stage 1.5 in ‘‘On’’ phase. The mini-mental state examination (MMSE) scored 30/30.

Another proband (Family 2, II:2) was a 51-year-old man who came to our hospital with rest tremor of bilateral hands. He complained about tremor and reduced agility for about 7 years. Initially, he was started on levodopa/benserazide 250 mg and piribedil 50 mg twice a day for 4 years. He developed postural instability at age 49, then he was switched to three times daily of the two medicines above. And dyskinesias were soon induced by levodopa. His other non-motor symptoms included RBD and hyposmia. At the time of assessment, his UPDRS-III score was 7/108 and H&Y stage was 1.5 in ‘‘On’’ phase. His MMSE test was 30/30.

# Supplementary Table 1. Primer sequences for Sanger sequencing.

| **Gene** | **Primer** | **Sequence (5’ to 3’)** | **Product size(bp)** |
| --- | --- | --- | --- |
| *TAF1* | TAF1-F | CACTTCCCCCTCCCTTTC | 294 |
|  | TAF1-R | CCCCTCACATCATCCAAGAC |  |

## Supplementary Table 2. Summary of *TAF1* rare variants within coding regions identified in the study.

| **Hg19 position** | **dbSNP ID** | **Ref** | **Alt** | **Protein**  **Alteration** | **Sequence**  **Ontology** | **ReVe** | **Male** | | **Female** | |
| --- | --- | --- | --- | --- | --- | --- | --- | --- | --- | --- |
|  |  |  |  |  |  |  | **No.**  **Case**  **(n=1,047)** | **No. Control**  **(n=795)** | **No.**  **Case**  **(n=870)** | **No.**  **Control**  **(n=857)** |
| X:70587376 | - | G | A | p.G50R | missense | 0.643:T | 0/1046 | 0/794 | 0/0/870 | 0/1/856 |
| X:70587397 | rs748677650 | C | T | p.L57L | synonymous | - | 2/1045 | 1/793 | 0/4/866 | 0/1/856 |
| X:70587414 | rs754759396 | G | A | p.T62T | synonymous | - | 0/1047 | 0/795 | 0/4/866 | 0/0/857 |
| X:70587949 | rs764028294 | A | G | p.N94S | missense | 0.098:T | 0/1047 | 1/794 | 0/0/870 | 0/2/855 |
| X:70595132 | rs5981106 | T | C | p.T156T | synonymous | - | 1/1046 | 0/782 | 0/0/870 | 0/0/857 |
| X:70596841 | - | G | T | p.A172S | missense | 0.475:T | 0/1047 | 0/795 | 0/1/869 | 0/0/857 |
| X:70597546 | rs28382158 | C | G | p.L270V | missense | 0.237:T | 0/1047 | 1/794 | 0/1/869 | 0/0/857 |
| X:70597572 | rs746812952 | G | A | p.E278E | synonymous | - | 2/1046 | 0/795 | 0/2/868 | 0/1/856 |
| X:70598123 | rs55836865 | A | G | p.Q324Q | synonymous | - | 0/1047 | 2/793 | 0/1/869 | 0/3/854 |
| X:70598170 | - | C | T | p.A340V | missense | 0.763:D | 0/1047 | 0/795 | 0/0/870 | 0/1/856 |
| X:70598183 | - | T | C | p.Y344Y | synonymous | - | 1/1046 | 1/794 | 0/0/870 | 0/0/857 |
| X:70598194 | rs925348346 | G | A | p.R348Q | missense | 0.137:T | 0/1046 | 0/795 | 0/0/870 | 0/1/856 |
| X:70598231 | - | C | T | p.G360G | synonymous | - | 0/1047 | 1/794 | 0/0/870 | 0/0/857 |
| X:70598256 | rs1265694938 | C | T | p.L369L | synonymous | - | 0/1047 | 0/795 | 0/1/869 | 0/0/857 |
| X:70598677 | - | T | C | p.F386L | missense | 0.103:T | 0/1041 | 1/794 | 0/0/870 | 0/0/857 |
| X:70598721 | rs773362861 | T | C | p.D400D | synonymous | - | 0/1047 | 0/795 | 0/0/870 | 0/3/854 |
| X:70598790 | - | A | G | p.K423K | synonymous | - | 0/1047 | 0/795 | 0/0/870 | 0/1/856 |
| X:70601671 | rs756449021 | G | A | p.R480H | missense | 0.800:D | 0/1043 | 0/792 | 0/1/869 | 0/0/857 |
| X:70601741 | - | T | A | p.L503L | synonymous | - | 0/1047 | 0/793 | 0/0/870 | 0/1/856 |
| X:70602697 | rs769418854 | C | T | p.T584T | synonymous | - | 0/1047 | 0/795 | 0/1/869 | 0/0/857 |
| X:70602909 | - | G | A | p.R614R | synonymous | - | 0/1036 | 0/789 | 0/0/870 | 0/1/856 |
| X:70602945 | rs1183038198 | T | C | p.F626F | synonymous | - | 0/1040 | 0/793 | 0/1/869 | 0/0/857 |
| X:70603868 | rs750096334 | A | G | p.T668T | synonymous | - | 1/1046 | 1/794 | 0/0/870 | 0/0/857 |
| X:70609515 | rs765090577 | A | G | p.E927E | synonymous | - | 0/1045 | 0/783 | 0/0/870 | 0/1/855 |
| X:70612511 | rs759265928 | G | A | p.T958T | synonymous | - | 1/1030 | 1/788 | 0/2/868 | 0/0/857 |
| X:70614004 | - | T | C | p.I1105I | synonymous | - | 1/1043 | 0/781 | 0/0/870 | 0/0/857 |
| X:70614048 | rs371501141 | G | A | p.R1120H | missense | 0.601:T | 0/1047 | 0/791 | 0/2/868 | 0/0/857 |
| X:70617155 | rs1314628439 | C | T | p.S1153S | synonymous | - | 1/1046 | 0/795 | 0/0/870 | 0/1/856 |
| X:70621455 | rs748405987 | G | A | p.A1288A | synonymous | - | 0/1047 | 0/795 | 0/1/869 | 0/0/857 |
| X:70626491 | rs755493949 | G | A | p.A1334A | synonymous | - | 0/1039 | 0/784 | 0/0/870 | 0/1/855 |
| X:70627477 | rs7050748 | G | A | p.S1387S | synonymous | - | 1/1046 | 0/795 | 0/0/870 | 0/0/857 |
| X:70627919 | rs781666863 | C | T | p.N1434N | synonymous | - | 0/1047 | 0/794 | 0/0/870 | 0/1/856 |
| X:70641163 | rs775139960 | A | G | p.P1463P | synonymous | - | 1/1045 | 0/786 | 0/0/870 | 0/0/857 |
| X:70643035 | rs758753633 | G | A | p.A1507A | synonymous | - | 1/1046 | 0/795 | 0/6/864 | 0/2/855 |
| X:70643893 | rs201667959 | A | G | p.M1549V | missense | 0.861:D | 0/1047 | 0/795 | 0/0/870 | 0/1/856 |
| X:70643907 | - | C | T | p.T1553T | synonymous | - | 0/1047 | 0/795 | 0/1/869 | 0/0/857 |
| X:70678147 | - | A | G | p.V1665V | synonymous | - | 0/1047 | 0/791 | 0/1/869 | 0/0/857 |
| X:70678168 | rs1020915271 | G | A | p.M1672I | missense | 0.139:T | 0/1047 | 1/793 | 0/0/870 | 0/0/857 |
| X:70679482 | rs1299642261 | G | A | p.E1715E | synonymous | - | 1/1044 | 0/794 | 0/1/869 | 0/1/856 |
| X:70680544 | rs776093280 | C | T | p.R1764C | missense | 0.376:T | 6/1041 | 0/795 | 0/4/866 | 0/4/853 |
| X:70680560 | rs147517498 | A | G | p.N1769S | missense | 0.092:T | 1/1046 | 0/795 | 0/0/870 | 0/0/857 |
| X:70683833 | rs1223812371 | C | T | p.D1853D | synonymous | - | 0/1047 | 0/795 | 0/2/868 | 0/0/857 |

n, sample size; Ref, reference allele; Alt, alteration allele.

The genotype counts were shown as hemizygous/wildtype for male individuals and homozygous/heterozygous/wildtype for female individuals.

**
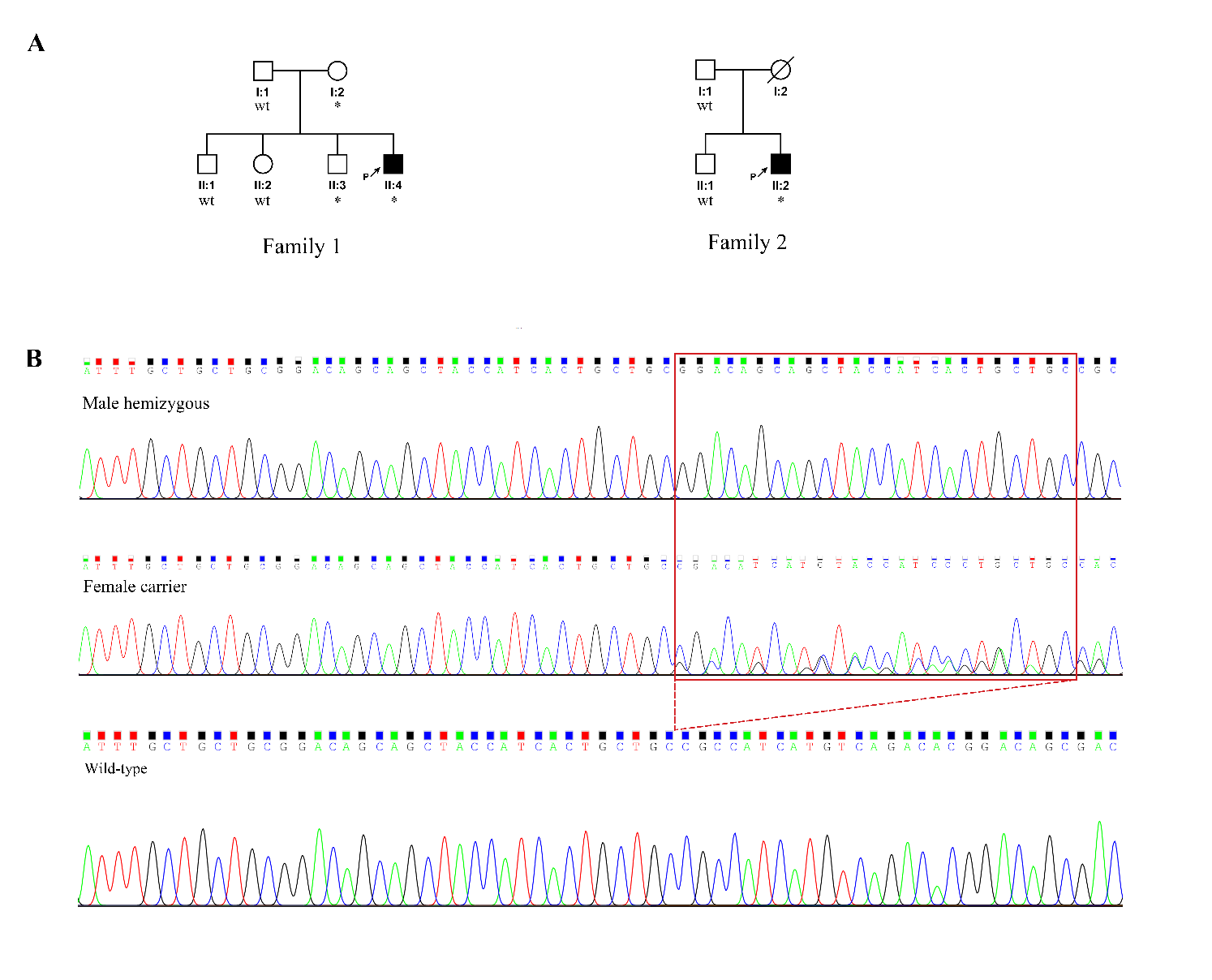
**

**Supplementary Figure 1.** Segregation analysis of the frameshift variant in *TAF1*. (A) Pedigree structure of the two families carrying the *TAF1* p.A19Dfs*50 variant is shown on the above (wt = wild-type, *=variant carrier). Arrows indicate the proband. Diagonal bars indicate deceased. Individuals with Parkinson's disease are indicated by solid squares. Unaffected individuals are indicated by open symbols. Circles, women. Squares, men. (B) Electropherograms shows the heterozygous and hemizygous state and wild-type control sequences.
